# Impact of Next Generation Sequencing on the Organization and Funding of Returning Research Results: Survey of Canadian Research Ethics Boards Members

**DOI:** 10.1371/journal.pone.0154965

**Published:** 2016-05-11

**Authors:** Iris Jaitovich Groisman, Beatrice Godard

**Affiliations:** Groupe de recherche Omics-Ethics, Institut de Recherche en Santé Publique, Université de Montréal, Montreal, Quebec, Canada; Renal Division, Peking University First Hospital, CHINA

## Abstract

Research Ethics Boards (REBs) are expected to evaluate protocols planning the use of Next Generation Sequencing technologies (NGS), assuring that any genomic finding will be properly managed. As Canadian REBs play a central role in the disclosure of such results, we deemed it important to examine the views and experience of REB members on the return of aggregated research results, individual research results (IRRs) and incidental findings (IFs) in current genomic research. With this intent, we carried out a web-based survey, which showed that 59.7% of respondents viewed the change from traditional sequencing to NGS as more than a technical substitution, and that 77% of respondents agreed on the importance of returning aggregated research results, the most compelling reasons being the recognition of participants’ contribution and increasing the awareness of scientific progress. As for IRRs specifically, 50% of respondents were in favour of conveying such information, even when they only indicated the probability that a condition may develop. Current regulations and risk to participants were considered equally important, and much more than financial costs, when considering the return of IRRs and IFs. Respondents indicated that the financial aspect of offering genetic counseling was the least important matter when assessing it as a requisite. Granting agencies were named as mainly responsible for funding, while the organizing and returning of IRRs and IFs belonged to researchers. However, views in these matters differ according to respondents’ experience. Our results draw attention to the need for improved guidance when considering the organizational and financial aspects of returning genetic research results, so as to better fulfill the ethical and moral principles that are to guide such undertakings.

## Introduction

The impact of communicating genetic individual research results (IRRs) to participants and their families is fairly well documented [1, 2]. Arguments that support or oppose returning IRRs have been widely discussed [3, 4]. The priority for research participants is to determine if they desire to receive personal information, and know about the risks, possible psychosocial problems, loss of privacy and insurability that may arise by obtaining such results [5, 6]. Concurrently, researchers and their institutions returning genetic IRRs need to foresee action plans to manage the consequences [7]. Returning Incidental Findings (IFs) either discovered by accident or during a deliberate search for particular genes [8] impact participants and institutions like returning IRRs does, with the addition of the “unexpected” factor. The wider use of Next Generation Sequencing (NGS), such as Whole Genome and Whole Exome Sequencing, is likely to produce all the more information of potential interest and clinical relevance for research participants. Returning IRRs and IFs resulting from NGS led to debates on a variety of issues [6, 9–11]. Whereas the costs of disclosing research results were examined well before the widespread use of NGS technologies [12], sharing NGS-generated data may prove challenging when wanting to ensure adequate care for a known or a newly discovered health condition.

Research Ethics Boards (REBs) evaluate protocols planning the use of NGS, assuring that any genomic finding will be properly managed [13]. In Canada, the national policy on the “Ethical Conduct for Research Involving Humans” (hereafter TCPS2)[14]–foresees that genetic research could reveal information that may be relevant for research participants and their families and thus, IRRs can be returned if they are validated and provide identification or prediction of a risk. [3, 15] As for IFs, TCPS2 considers them to be “material incidental findings” if they have significant benefits for a research participant [14]. Canadian researchers funded by any of the three Canadian research granting agencies must comply with TCPS2. Hence, they are expected to plan how they will manage IRRs and IFs, and communicate with their REBs in relation to potential results and their strategy to convey information to participants. Consequently, REB members play a central role in the disclosure of such information, assuring that the principles of autonomy, beneficence, and justice be applied in returning genetic research results. Therefore, we deemed it important to examine the views of Canadian REB members on the use of NGS in research. With this intent and based on current literature we developed a web-based survey addressing subjects related to the return of IRRs and IFs [6, 16–20]. We report herein our results on REB members’ opinions regarding the organizational and financial aspects of such endeavours.

## Materials and Methods

### Sample and Recruitment

We invited Canadian REB members to participate in an anonymous, web-based survey by sending a letter of invitation—containing a link to the questionnaire—to REB coordinators and REB chairs whose contact information was publicly available, asking them to distribute the invitation among their Boards’ voting members. The Canadian Association of Research Ethics Boards (CAREB) and the Canadian Association of Research Administrators (CARA) distributed the invitation among their associates via email. In all scenarios, reminder emails were sent twice, about two weeks apart.

An invitation to participate was also posted on these associations’ LinkedIn sites, as well as on the Quebec Ministry of Health, Ethics and Quality Directorate website. The invitation letter and survey were available in English and in French. At the end of the survey responders were requested to forward the invitation to other REB members, so as to increase the number of participants. The first invitation was sent at the end of April 2014, and the survey was available until the end of August 2014.

### Survey Development and Data Collection

To inform survey development, we used results from our previous work on NGS in mental health and brain disorder research, and on current Canadian approaches to consent for genetic/genomic research [14, 19–21]. We also considered the results of related studies, conducted elsewhere, among REB members and researchers, and subjects that surfaced in current literature regarding the recourse to NGS in research settings [6, 8, 16, 17, 22]. We described Individual Research Results (IRRs) as the information about a research participant generated in the course of genomic research. Information with potential health and/or reproductive importance and which is of personal relevance, yet discovered outside the original purpose of the study, was defined as an Incidental Finding (IF) [4]. Because the TCPS2 requires that research results be validated if they are to be returned, we informed survey participants to keep in mind that for every question on the return of IRRs and / or IFs, we were referring to validated genetic results [14].

The survey contained six themes covered by multiple choice questions in addition to demographic information of participants (S1 Questionnaire). It took approximately 30 minutes to complete. Responses including unique variables increasing the likelihood of participant identification were withdrawn, just as geographical location and role of board member and/or board characteristics were not combined. This study and all the accompanying documents were approved by the Université de Montréal Health Science Research Ethics Board.

The TCPS2 (2010) was updated to TCPS2 (2014) after our survey was closed and the analysis was underway. The questions presented in the survey are not related to the changes presented in TCPS latest version, and the updates do not influence our analysis [14].

### Statistical Analysis

Descriptive statistics and cross-tabulation Χ^2^ analysis produced a portrait of REB members’ opinions and views on the use of NGS in research, the return of aggregated and individual results, as well as incidental findings. Chi square analysis allowed us to draw comparisons between respondents according to their answers and characteristics (e.g., position in an REB). We considered a P value of 0.05 or less as statistically significant. Data were analyzed using SPSS version 22 (IBM, Somers, NY). Finally, sample sizes varied by question because participants were allowed to skip any question they did not wish to answer. Furthermore, we specify the sample size for each question for clarity. When the sample was small for Χ^2^ analysis, further analysis was conducted using the likelihood ratio to confirm the significance of particular results.

## Results

### Sample

Eighty-one REB members enrolled (Table 1). The majority of participants (66.7%, N = 54/81) declared having experience in conducting research with human subjects, and 27.5% (N = 14/54) of them in handling genetic/genomic research.

**Table 1:** Participants’ Characteristics.

Position on an REB (n = 76)*	n	%
Chair	16	21.1
Jurist/ Ethicist	11	14.5
Member of the community	14	18.4
Scientific member	20	26.3
Other	15	19.7
**Years of experience in an REB (n = 81)**		
0 to 4 years	35	43.2
5 to 9 years	27	33.3
10 years ++	19	23.5
**Experience conducting research with Human participants (as per TCPS2)**		
Yes (n = 81)	54	66.7
If Yes, Genetic research (n = 51)	14	27.5
**Work setting**		
Hospital	39	31.0
Academic Medical Centre	25	19.8
University	36	27.8
Government	12	9.5
Private	9	6.3
Other	7	5.6
**Type of research reviewed (Note, percentages do not total 100% because respondents could check all that apply) (n = 80)**		
Social Sciences	54	67.5
Behavioral Sciences	47	58.8
Medical/Health	75	93.8
**Number of new protocols reviewed/year (n = 79)**		
0–49	43	54.4
50-over	36	45.6
**Revision of genetic/genomic protocols (n = 79)**		
Many times	27	34.2
A few times	31	39.2
Rarely / Never	21	26.6

*REB stands for Research Ethics Boards; n refers to number of respondents

### Main Findings

#### NGS and participants’ protection

REB members participating in our survey considered that for the purpose of “informed” consent, research participants needed to receive further explanations on DNA sequencing when there were plans to use NGS. More than half of the respondents viewed the change from traditional sequencing to NGS as more than a technical substitution (Table 2). Furthermore, they believed that if DNA samples were collected for traditional sequencing, a subsequent sequencing using NGS technologies required an explanation to participants (81.7%, N = 58/71), mostly through a new consent form. This explanation was considered relevant to the description of risks and benefits that research participants should take into account so as to make an informed decision (Table 2).

**Table 2:** Views on Participation in NGS* Research.

For the purpose of informed consent, change from traditional sequencing to NGS is… (n = 77)	n*	%
A technical change that does not require the addition of specific explanations	4	5.2
A technical change that requires the addition of specific explanations	27	35.1
Not a mere technical change and requires the addition of specific explanations	46	59.7
**For DNA samples collected for traditional sequencing, does its subsequent use requires an explanation to participants? (n = 71)**		
No	4	5.6
Yes	58	81.7
Other	9	12.7
**If yes, does the subsequent use of NGS on such samples require providing an explanation? (n = 57) (respondents could check all that apply)**		
Through a new consent	51	87.9
Through a letter of information	18	31.0
Other	4	7.0
If yes, do you consider that explaining the use of NGS on either newly acquired or previously collected samples is relevant to the description of benefits and risks that research participants should consider in order to make an informed decision? (N = 70)*		
Checked	67	94.4
Other	3	4.2

*Participants could check could check all that apply and could also mark “other” if they thought something different to the statement; NGS stands for Next Generation Sequencing Technologies; n refers to number of respondents

#### Return of research results

Seventy seven percent of respondents (N = 54/70) agreed on the importance of returning aggregated research results. The most compelling reasons were recognizing participants’ contributions, benefiting participants by increasing their awareness of scientific progress, and promoting good research practices. The least important was to promote researchers’ and institutions’ scientific reputation (Table 3). A 37% of respondents (N = 25/67) have experience evaluating protocols for genetic/genomic studies offering return of aggregated results.

**Table 3:** Aggregated Research Results.

Is it important to return aggregated results? (n* **= 70)**	**n**		**%**	
Yes	54		77.1	
No	8		11.4	
I don’t know	5		7.1	
Other	3		4.3	
**Which is important when deciding whether or not to return aggregated research results?**	**n**	**Yes** (%)	**No** (%)	**I don’t know** (%)
Recognizing participants contribution	69	82.7	14.5	2.9
Limiting risks of identifying participants	65	75.4	16.9	7.7
Benefiting participants by increasing awareness of scientific progress	67	83.6	9.0	7.5
Facilitating contact with and future recruitment of participants	64	50.0	34.4	15.6
Promoting good research practices	67	82.1	16.4	1.5
Promoting researchers’ and institutions’ scientific reputation	63	49.2	39.7	11.1
**Do you evaluate/have you evaluated protocols for genetic/genomic studies offering return of aggregated results?** (n = 67)	**n**		**%**	
Yes	25		37.3	
No	42		62.7	

*n refers to number of respondents

Thirty five percent of respondents (N = 23/65) reported they have experience evaluating projects involving returning genetic IRRs (Table 4). When evaluating the provision of genetic IRRs, current regulations on the conduct of research with human participants and the risks the latter could be exposed to, were more relevant for our respondents than the financial costs involved in returning such results (Table 4). Recognizing participants’ contribution was not a priority for REB members when it came to conveying IRRs (Table 4). When IRRs reveal the genetic causes of a condition and/or a specific response to medication, the majority of respondents agreed on providing them. When IRRs only indicated a *probable medical condition*–or a *probable explanation about response to medication*, fifty percent (N = 30/60) of respondents were in favour of conveying such information (Table 4). When asked about experience in conducting genetic/genomics research, thirty respondents declared they did not have it (N = 30/40). However, they were significantly more prone to convey information indicating probability of a condition (66.7% p< 0.042).

**Table 4:** Individual Research Results.

Which of the following is important when evaluating whether or not IRRs* **should be returned?**	n*	**Yes (%)**	**No (%)**	**I don’t know (%)**
Recognizing participants’ contribution	66	62.1	36.4	1.5
Current regulations (Nat. Prov. Inst.)	68	77.9	13.2	8.8
Risk to participants	68	95.6	4.4	
Risk of stigma	67	77.6	19.4	3
Risk of increased stress	68	92.6	4.4	2.9
Risk of affecting participants’ family relations	67	85.1	10.4	4.5
Financial costs	59	42.4	57.6	
Future health	65	97.0	3.0	
**Type of IRRs**	**n**	**Yes (%)**	**No (%)**	
**Results explain**		82.5	17.5	
Genetic causes	61	82.0	18.0	
Response to medication	61	80.3	19.7	
**Results indicate probability**		51.6	48.4	
Genetic causes	60	50.0	50.0	
Response to medication	60	48.3	51.7	
**Experience in returning IRRs**	65	Yes (%)		
Yes		35.4		
No		64.6		

*IRRs stands for Individual Research Results, defined as those related to the study’s subject matter; n refers to number of respondents

When assessing whether or not IFs should be returned, respondents felt that recognizing participants’ contribution was not a priority (Table 5). Current regulations and risks to participants were equally important, much more so than the financial cost of communicating IFs (Table 5). Responses to our questions on returning IFs were also influenced by the severity and preventability of a condition (Table 6). For a life-threatening one, respondents were more inclined to return an IF if the condition was preventable. However, about 50% of respondents (N = 39/67) would still accept communicating an IF even if the condition were not preventable. For those in favour of conveying IFs independently of severity and preventability, their responses were not influenced by the age of research participants (from childhood to adulthood), or by age of onset (early age to late in life) (S1 Table). The higher the chance an individual participant had of developing a serious health condition—that could be prevented—the more REB members were inclined to accept conveying the information when it was obtained incidentally, and this, independently of age of onset (S2 Table). From the 60 respondents that answered questions on IFs, 40% (N = 24/60) had experience in evaluating protocols that involved the communication of incidental findings.

**Table 5:** Return of IFs*—what to consider.

Which of the following is important when evaluating whether or not IFs should be returned?	n*	Yes (%)	n	No (%)	n	I don’t know (%)
Recognizing participants’ contribution	28	43.8	34	53.1	2	3.1
Current regulations (Nat. Prov. Inst.)	51	77.3	12	18.2	3	4.5
Risks to participants	60	92.3	5	7.7		
Stigma	49	76.6	14	21.9	1	1.6
Increased stress	59	90.8	5	7.7	1	1.5
Affecting participants’ family relations	52	80	9	13.8	4	6.2
Financial costs	24	41.4	34	58.6		
For researchers	23	36.5	34	54	6	9.5
For institutions	18	28.1	39	60.9	7	10.9
Future health	62	96.9	2	3.1		
Results contributing to participants’ reproductive choices	58	89.2	6	9.2	1	1.5
Type of results in relation to severity of condition	57	90.5	4	6.3	2	3.2

*IFs stands for Incidental Findings; n refers to number of respondents

**Table 6:** Return of IFs*—type of condition.

IF a LIFE THREATENING condition CANNOT be prevented… Do you agree with offering research participants the communication of incidental findings for this type of condition? (n = 67)	n	%
**Yes**	39	58.2
**No**	16	23.9
**I don’t know**	12	17.9
**IF a LIFE-THREATENING condition CAN be prevented**… Do you agree with offering research participants the communication of incidental findings for this type of condition? (n = 66)		
**Yes**	63	95.5
**No**	2	3
**I don’t know**	1	1.5
**IF a NON-LIFE-THREATENING condition CANNOT be prevented…** Do you agree with offering research participants the communication of incidental findings for this type of condition? (n = 64)		
**Yes**	34	53.1
**No**	19	29.7
**I don’t know**	11	17.2
**IF a NON-LIFE-THREATENING condition CAN be prevented…** Do you agree with offering research participants the communication of incidental findings for this type of condition? (n = 61)		
**Yes**	53	86.9
**No**	6	9.8
**I don’t know**	2	3.3

*IFs stands for Incidental Findings; n refers to number of respondents

#### Genetic counseling

Fifty percent (N = 57) of those responding on the issue of genetic counseling indicated that their REB required researchers to offer genetic counseling in genetic/genomic projects that planned to return IRRs and/or IFs (S3 Table). Close to 60% (N = 34/55) also noted that researchers at their institutions were asked to include an explanation about the provision of genetic counseling on the consent form. The provision of an explanation about genetic counseling is important for an informed consent for the majority of the 56 respondents to this question (91.1%, N = 51/56). When asked if there institutions facilitate the provision of genetic counselling, there were 31 respondents out of 41 that answered “yes”.

Respondents indicated that offering genetic counseling was very important to protect research participants’ future health and mitigate risks, as were clear explanations on genetic counseling. Less important was the recognition of participants’ contribution to research, and even less so the financial aspect of providing genetic counseling.

#### Organizational and financial aspects of returning research results

When we asked REB members whom, from their perspective, should fund, organize and be responsible for communicating genetic/genomic IRRs and IFs (Table 7), granting agencies were named by 65% (N = 30/46) as mainly responsible for funding, while organizing, returning and conveying IRRs and IFs lay on the shoulders of researchers (72.9%, N = 35/48).

**Table 7:** Management of return of IRRs and IFs*.

**Management of return of IRRs**						
	**Funding (n = 46)**		**Organization (n = 45)**		**Return of results (n = 48)**	
	**n**	**%**	**n**	**%**	**n**	**%**
Institution	14	30.4	14	31.1	11	23.4
Granting Agency	30	65.2	12	26.7	12	25.5
Research Team	20	43.5	35	77.8	35	72.9
REB	1	2.2	6	13.3	0	0.0
I don’t know	1	2.2	1	2.2	1	2.1
Other	4	8.7	3	6.7	7	14.9
**Management of return of IFs**						
	**Funding (n = 45)**		**Organization (n = 48)**		**Return of results (n = 47)**	
	n	%	n	%	n	%
Institution	15	33.3	15	32.6	12	25.5
Granting Agency	29	64.4	11	23.9	10	21.3
Research Team	19	42.2	36	78.3	35	74.5
REB	1	2.2	6	13.0	0	0.0
I don’t know	1	2.2	1	2.2	1	2.1
Other	4	8.9	3	6.5	7	14.9

*IRRs refers to Individual Research Results, IFs to Incidental Findings and n to number of respondents

Position on a REB, familiarity with evaluating genetic/genomic studies offering return of IRRs and/or IFs, and experience in conducting research protocols with human participants involving genetic tests were the main factors that influenced REB members to take financial costs into consideration when evaluating if IRRs and IFs should be returned to participants. The results for IRRs and IFs were similar (S4 Table). Among REB members, the least inclined to consider financial costs when evaluating genetic/genomic projects planning to return IRRs and IFs were members of the community (For IRRs: 11% N = 58 p = 0.018, and for IFs: 0% N = 57 p< 0.00). Members with experience evaluating genetic/genomic studies offering return of results considered financial costs differently from those without that experience. Respondents that have some experience reviewing genetic / genomic protocols consider financial costs important at time of returning IRRs (52% N = 58 p = 0.02). Respondents that have some experience evaluating protocols that involve returning of IFs also considered financial costs of conveying such results important (91.7% N = 57 p = 0.026). REB members that were not themselves researchers considered that it was the research team’s responsibility to return results (92.6% N = 31 p = 0.015). As for funding, REB members that had some experience reviewing genetic/genomic research felt it was the research team’s responsibility to fund this endeavour (60% N = 45 p = 0.030).

## Discussion

In Canada, revisions to the TCPS2 include open consultations conferring a voice to the wider research community. However, individual REB members’ opinions may not coincide with TCPS2 requirements.

In concordance with current discourse, more than half of participants agreed that changes in technology with the potential to retrospectively impact participants’ preferences—such as the case of traditional sequencing being replaced by NGS—should be brought to their attention [18, 20, 23]. For these respondents, conveying such information should be done mainly through a new consent form. Such practice calls on new requirements in the consent process, imposed either by individual REBs or as a result of institutional regulations.

For participants that responded questions on returning aggregate genetic research results, the main reasons behind providing this information are recognizing participants’ contribution and promoting good research practices, as expressed elsewhere [24]. However, according to our data and as previously reported, this is still an uncommon practice [24]. Discussion on the provision of these outcomes in genetic research has been somehow sidelined by the exciting possibilities of informing research participants about genetic IRRs and/or IFs. We agree that returning aggregate research results is independent of, and it does not substitute the obligations of returning IRRs and/or IFs [24].

Some research protocols are designed to return IRRs, while in others, the return of IRRs is not considered among the goals of the project. For our sample of Canadian REB members, the main factors leading to approval or refusal to allow the return of IRRs are the risks and health benefits to participants. Less important is the recognition of participation in a research project, in opposition to their stand on returning aggregated research results. Earlier work in the matter of returning genetic results derived on recommendations to consider the resources needed to support the process [6]. However, our respondents considered the financial aspects of returning IRRs the least important. The TCPS2 indicates that IRRs *have to be returned* if they identify or predict a risk [14]. Yet, clear regulations should be set out based not only on the benefits and risks to participants but the availability of resources allocated to the actual return of results.

In Canada, the role given to REBs by the TCPS2 is fundamental to the approval of returning IFs [14]. Boards thus have to deal with details both obvious and subtle to define what, when and how such information is to be conveyed. About half of the 50 to 60 respondents to the questions on IFs would accept communicating them even if the condition/s were not preventable. Notably, 40% of those completing questions on IFs had experience evaluating protocols that included the communication of these results. It should be noted that the Network of Applied Genetic Medicine of Québec through their Statement of Principles emphasizes that parents should not—cannot—refuse receiving genetic information about a condition affecting their children that was detected incidentally in a research setting and that will develop at an early age in the life of their offspring [25]. To guarantee disclosure there should be an “effective” and “preventive” treatment for such a condition in childhood and adolescence [25]. Thus, REBs and researchers in the Province of Quebec have to elaborate proper plans—budget, human resources -, to accomplish such a task before a project is approved. Minors are considered a vulnerable population, yet a similar approach on conveying IFs to adults could be perceived as paternalistic [26]. In our survey, when respondents were asked about returning information for non-preventable conditions, some marked the option “I don’t know”, which illustrates the ethical dilemma existing when the risks and benefits for participants are not clear. Interestingly, REB chairs recruited from institutions conducting genome-wide association studies completing an open-ended questionnaire expressed their preference for “policies where disclosure procedures would be determined prior to approval of the research”. However, researchers from the same institutions participating in the same study favoured policies with “a case-by-case determination regarding whether or not genetic IF disclosure would be offered after discovery” [22]. A recent survey among members of the public, genomic researchers and health professionals showed that IFs should be made available to research participants. However, respondents expressed that they did not expect researchers to actively search for incidental findings in research settings [27]. Further empirical studies evaluating the feasibility on conveying IFs, and research participants’ expectations in this matter would help identify what stakeholders need and require in the return of IFs.

For Canadian researchers, the provision of genetic counseling to communicate genetic research results is considered as a needed resource by the TCPS2 [14]. Respondents to the questions on genetic counseling contemplated the ethical issues surrounding its provision, namely the protection of human participants in genetic research. The number of respondents that addressed the matter of institutions providing genetic counseling was low. At the same time the financial aspects of providing genetic counseling were not so important for the REB members participating in our survey. There seems to be a disconnect between what has to be provided and how to put it into action. If genetic counseling is not provided because of financial or organizational constraints, it loses its value as a means of protecting research participants and their families. Further empirical work is needed to determine if and how institutions hosting genetic research endeavors help on providing genetic counseling.

While the validity of research results, the ethical debates surrounding the return of IRRs and IFs, and the impact on research participants have been the subject of intense debate, it seems to us that basic organizational aspects have been somehow overlooked. The sample of Canadian REB members participating in our study emphasized the role of granting agencies in financial support, and of researchers in organizing and conveying IRRs and IFs. Analyses of the costs of returning research results before the wider use of NGS included the costs of study, software design, the extra effort required to generate individual-level results, the complexity of maintaining confidentiality, costs of the disclosure procedure itself and the possible need for counseling [12]. While it could be considered that there is no effort to obtain results in current genetic research, the rise in data obtained from NGS accompanies the increased costs of all the above listed activities. There are subsequent expenses when returning genetic results, namely the costs of therapeutic and/or prevention treatments, which affect genetic/genomic studies [28]. The inequalities of health care provision in terms of type of health care system—or lack thereof—strengthens then the need to foresee more than the costs of counseling, softwares and confidentiality. The sample of REB members surveyed shows that position in a REB, familiarity with evaluating genetic/genomic studies offering return of IRRs and/or IFs, and experience in conducting research protocols with human participants involving genetic tests make REB members aware of the financial costs of returning genetic results. However, being concerned and informed is not in and of itself sufficient to move closer towards a fulfillment of the principles of beneficence and justice, as discussed by Dal-Re et al [13].

### Limits

Our study included the opinions of a restricted number of participants and may not be representative of the Canadian REB community. The limited number of respondents on the matter of age of research participants and age of onset of a given condition, the severity and the preventability of a health condition, and on other issues addressed in our survey calls to further explore the opinions of REB members and other stakeholders on the returning of genetic research results. The length of our questionnaire as well as the complexity of the topics might have been a deterrent to many. We also understand that many REB members only evaluate social and behavioral studies and might have decided not to express their views on subjects for which they lack professional experience.

## Conclusion

The present study allowed us to identify how much—or how little—Canadian REB members responding to our survey pay attention to the organizational aspects of returning research results, including its financial aspects. We were also able to recognize the lack of clear rules on distributing funds for the various activities implied in returning results, as well as the workload assigned to research teams and REBs with regulations that are not clear enough to provide a sound framework.

While the number of participants to our survey is limited, this study draws attention to the need for guidance to ensure that the organizational aspects of returning genetic research results are considered, so that researchers can fulfil their projects and REB members can guarantee research participants protection in this process. Empirical research on the organization of returning genetic research results, including the manpower to conduct the research as well as to convey information and offer counseling, if and when needed, the distribution of the financial costs of producing validated genetic data, as well as the anticipation of unforeseen health costs for newly discovered conditions, will greatly contribute to fulfilling the ethical and moral principles that should guide the return of genetic results.

## Supporting Information

S1 Questionnaire Survey presented to Canadian REB members. (DOCX)

S1 Table Conveying Genetic Incidental Findings: age of onset and age of participants and its relation to the severity of a health condition—Part I. (DOCX)

S2 Table Conveying Genetic Incidental Findings: age of onset and its relation to the probability of a health condition—Part II. (DOCX)

S3 Table Genetic Counseling. (DOCX)

S4 Table Financial costs of returning IRRs and IFs and the role of REB members. (DOCX)
