# Supplementary material for: Impact of Next Generation Sequencing on the Organization and Funding of Returning Research Results: Survey of Canadian Research Ethics Boards Members
Source: PLoS One. 2016 May 11;11(5):e0154965. doi: 10.1371/journal.pone.0154965 (PMC4868059; doi:10.1371/journal.pone.0154965)
Supplement: S1 Questionnaire — (DOCX) [file pone.0154965.s001.docx]

THE FOLLOWING QUESTIONS REFER TO YOU AS A REB MEMBER

***1: Are you or have you been a REB member? Yes/No***

If No: Could you please forward the invitation to participate in this survey to REB members you may know?

If yes: survey follow

***1 - REB MEMBERSHIP***

TO BROWSE THIS SURVEY USE THE TABS ON TOP OF THE PAGE.

***1.1***: What position(s) do you now hold or have ever held on a REB?

please, mark all that apply Present/Past/N/A

Chair / Ethicist / Jurist / Member of the community / Scientific member / Other

***1.2***: How long have you been a REB member?

***1.3***: Are you currently conducting or have you ever conducted research with human participants based on the (TCPS2) definition of research : an undertaking intended to extend knowledge through a disciplined inquiry or systematic investigation?

Yes / No

Is / was it related to genetics/genomics?

Yes / No

***REB SETTINGS AND ACTIVITIES***

***1.4***: Work setting (please, mark all that apply) Present / Past /N/A

Hospital / University / Academic Medical Center / Government / Private / Other

***1.5***: What kind of research have you reviewed as a REB member? (Please, mark all that apply)

Social sciences / Behavioural sciences / Medical / health / Other

***1.6***: How many research protocols (without considering protocol amendments or annual reviews), do you currently review per year? approximate number during the last year

***1.7***: How frequently do you review or did you ever review protocols involving genetics/genomics?

Many times A few times Rarely Never

***2 - YOUR VIEWS ON PARTICIPATION IN NGS RESEARCH***

As we explained to you on the invitation and consent letter, one of the main advantages of NGS is its ability to produce a much greater volume of genetic information than traditional sequencing, at an accessible cost. At the same time, it introduces new challenges such as data storage and sharing, interpretation and potential clinical significance of results, and impact of unexpected information to participants, among others.

***2.1***: For the purpose of an informed consent, do you consider the change from traditional sequencing to NGS to be:

1. A technical change in study procedures that does not require the addition of specific explanations in the consent form
2. A technical change in study procedures that requires the addition of specific explanations in the consent form
3. Not a mere technical change in study procedures and that requires the addition of specific explanations in the consent form

Comments:

If b or c are marked:

***2.2***: Many NGS studies use DNA samples that were initially collected for traditional sequencing: does the subsequent use of NGS on such samples require providing an explanation to participants?

Yes / No / Other

***2.3***: Do you consider that explaining the use of NGS on either newly acquired or previously collected samples is relevant to the description of benefits and risks that research participants should consider in order to make an informed decision?

Yes / No / Other

If yes:

a. Through a new consent

b. Through a letter of information

c. Other. Please, explain:

***2.4***: Application of NGS technologies often calls for sharing genetic data through multiple databases. Recent empirical research shows participants prefer a restricted release of data to publicly accessible databases.

Do you consider it is appropriate to offer participants different options as to how their genetic data is shared?

Yes

No

***2.5***: From your experience/point of view, how likely it is that a study participant whose genetic data has been coded could be

personally identified by a third unauthorized party?

harmed as a result of identification by a third unauthorized party?

Options: Certain / Likely / Unlikely / Impossible / I don’t know

***3 - RETURN OF RESULTS TO RESEARCH PARTICIPANTS***

AGGREGATED RESULTS, INDIVIDUAL RESEARCH RESULTS AND INCIDENTAL FINDINGS IN GENETIC / GENOMIC STUDIES USING NGS TECHNOLOGIES

AGGREGATED RESULTS

Aggregated results are the overall results of a study, which relate to participants in the study as a group.

***3.1***: In your opinion, is it important to return aggregated research results to participants?

Yes / No / I don't know / Other

***3.2***: Which of the following is important to you when evaluating whether or not aggregated research results should be returned to participants? Yes No I don't know

Recognizing participants’ contribution

Limiting risks of identifying participants

Benefiting participants by increasing awareness of scientific progress

Facilitating contact with and future recruitment of participants

Promoting good research practices (i.e. reporting and disseminating research)

Promoting researchers’ and institutions’ scientific reputation

Other: (Please, explain)

Comments:

***3.3***: Do you evaluate/have you evaluated protocols for genetic / genomic studies offering return of aggregated results? Yes No

***3.4***: For studies addressing mental health disorders or brain disorders as examples of stigmatizing conditions, would your position on returning aggregated research results of a genetic/genomic nature differ? Yes / No Please explain:

Which of the following do you think would impact your position? (please, mark all that apply)

a) Potential increase risk of participants’ stigmatization

b) Potential increase of participants’ vulnerability

c) Other

INDIVIDUAL RESEARCH RESULTS

Individual research results are results directly related to the subject that prompted the study (for instance, individual results about the genetic causes of the disease under study). Where applicable, please consider that results meet criteria of scientific and clinical validity.

***3.5***: Which of the following is important to you when evaluating whether or not individual research results should be returned to participants? Yes /No / I don't know

Recognition of participants’ contribution

Current regulations (National, Provincial, Institutional)

Risk of stigma to participants

Risk of increasing participants’ levels of stress or anxiety

Risk of affecting participants’ family relations

Financial costs for researchers of returning individual research results

Financial costs for Institutions of returning individual research results

Results contributing to participants’ informed reproductive choices

Type of results in relation to the severity of the condition (for instance life-threatening, serious non-threatening, non-serious, preventable or not)

Other: (Please, explain)

Comments:

***3.6***: Which of the following types of individual research results, classified according to their significance for a studied disease do you think should be offered to be returned to participants? Yes / No

Results explaining the genetic cause(s) of the studied disease

Results indicating only the PROBABLE genetic cause(s) of the studied disease

Results explaining genetic cause(s) of the response to the medications use to treat the studied disease

Results indicating only the PROBABLE genetic cause(s) of the response to the medications use to treat the studied disease

Comments:

***3.7***: Do you evaluate/have you evaluated protocols for genetic / genomic studies offering return of individual research results? Yes / No

***3.8***: For studies addressing mental health disorders or brain disorders as examples of stigmatizing conditions, would your position on returning individual research results of a genetic/genomic nature differ? Yes / No

Please explain:

Which of the following do you think would impact your position. (please, mark all that apply)

a) Potential increase risk of participants’ stigmatization

b) Potential increase of participants’ vulnerability

c) Other

INCIDENTAL FINDINGS

NGS approach has a high likelihood of discovering unanticipated results (incidental findings) that can predict a clinical condition unrelated to the primary goal of the study. Returning incidental findings to research participants could be perceived both as a risk and as a benefit to them. Where applicable, please consider that results meet criteria of scientific and clinical validity.

***3.9***: Which of the following is important to you when evaluating whether or not incidental findings should be returned to participants? Yes No I don't know

Recognition of participants’ contribution

Current regulations (National, Provincial, Institutional)

Risk of stigma to participants

Risk of increasing participants’ levels of stress or anxiety

Risk of affecting participants’ family relations

Financial costs for researchers of returning individual research results

Financial costs for Institution of returning individual research results

Results contributing to participants’ informed reproductive choices

Type of incidental findings in relation to the severity of the condition (for instance life-threatening, serious non-threatening, non-serious, preventable or not)

Other: (Please, explain)

Comments:

Please respond if you agree or not with offering research participants the communication of Incidental Findings, and if yes under which circumstances:

***3.10.1***: a) Incidental findings that address a life-threatening condition that cannot be prevented

Yes / No / I don't know

***3.10.2***: b) Incidental findings that address a life-threatening condition that can be prevented

Yes / No / I don't know

***3.10.3***: c) Incidental findings that address a serious but not life-threatening condition that cannot be prevented

Yes / No / I don't know

***3.10.4***: d) Incidental findings that address a serious but not life-threatening condition that can be prevented

Yes / No / I don't know

***For question 3.10.1 to 3.10.4 If answer is Yes***

If you agree to returning incidental findings for a serious but not life-threatening condition that can be prevented, for which age of onset would you consider it appropriate?

(please mark all that apply)

Participant is young (child to young adult) // Participant is adult

Condition has:

Early age of onset

Onset in young adulthood (20 to 34 years of age)

Onset in adulthood (35 to 54 years of age)

Late age of onset (55 years of age and over)

Comments:

***3.11***: Suppose it were possible to return incidental findings about a serious condition that could be prevented. Depending on the chance of having this condition, in which case do you think these results should be offered to participants?

(Please, mark all that apply)

Early age (childhood, adolescence) /Young adulthood (20 à 34 years of age) / Adulthood (35 à 54 years of age) / Late in life (55 years of age and over) /No / I don't know

A 1% chance_A 10% chance_A 50% chance_A 90% chance_Comments:

***3.12***: Do you evaluate/have you evaluated protocols for genetic / genomic studies offering return of incidental findings? Yes/ No

***3.13***: For studies addressing mental health disorders or brain disorders as examples of stigmatizing conditions, would your position on returning incidental findings of a genetic/genomic nature differ?

Yes / No / Please explain:

Which of the following do you think would impact your position. (please, mark all that apply)

a) Potential increase risk of participants’ stigmatization

b) Potential increase of participants’ vulnerability

c) Other

***4 - CONDITIONS SURROUNDING THE RETURN OF RESULTS***

VIEWS ON THE CONDITIONS SURROUNDING THE RETURN OF GENETIC INDIVIDUAL RESULTS AND /OR INCIDENTAL FINDINGS

***4.1***: Does your current or former REB require that researchers offer genetic counselling in their genetics/genomics research protocols when planning the return of individual research results or incidental findings? Yes / No / Other

***4.2***: Are researchers at your current and/ or past institution(s) required to include an explanation on the provision of genetic counselling on the consent forms? Yes / No / Other

***4.3***: Do you consider an explanation on the provision of genetic counselling to be important for participants’ informed consent? Yes / No

It is generally accepted that professionals with proper training (i.e. genetic counsellors, nurses involved in genetic services, medical geneticists) are responsible for communicating / explaining genetic study results.

***4.4***: Is/are the institution(s) to which your current(s) / past REB(s) belong, helping to make these human resources available to the researchers? Yes / No / Other

***4.5***: Which of the following considerations are important when offering genetic counselling to research participants? Yes /No / I don't know

Explicit mention in the consent form of eligibility criteria for receiving genetic counselling

Results predicting a clinical condition (clinically validated results)

Results indicating a chance (probability) of a clinical condition

Age of onset of the condition

Results contributing to participants’ informed reproductive choices

Recognition of participants’ contribution to research

Risk of stigma to participants

Risk of increasing participants’ levels of stress or anxiety

Risk of affecting participants’ family relations

Possibility to offer genetic counselling to first degree relative of an affected research participant

Financial costs for researchers

Financial costs for Institution

Other: (Please, explain)

Comments:

***5 - YOUR VIEWS ON MODALITIES FOR RECONTACTING PARTICIPANTS***

***5.1***: Researchers may need to recontact participants. For which of the following reasons do you consider it important to ask for permission to recontact participants at time of consent? (Please, mark all that apply) Yes / No / I don't know

For secondary use of samples

For secondary use of data

To obtain further health information

To obtain more samples

To return individual research results

To provide information on incidental findings

For genotype driven recruitment (recruitment based on initial genetic research results even if there is no clear significance)

For recruitment for related or unrelated studies

Other: (Please, explain)

Comments:

***5.2***: Do you agree with the following statement: Researchers should be able to recontact participants in all cases provided that participants be free to refuse to follow up on this recontact. Yes / No / Comments:

***5.3***: How often have you come across researchers that have approached a REB to define a strategy for recontacting participants?

Many times A few times Rarely Never I don’t know

Any type of research

Genetics/Genomics research

Comments:

***6 - SECONDARY USE OF MATERIAL AND OF THE GENERATED GENETIC DATA***

THE MATTERS OF SECONDARY USE AND SHARING OF MATERIAL AND GENERATED GENETIC DATA HAVE BECOME KEY ELEMENTS IN THE DEVELOPMENT OF GENETIC/GENOMIC RESEARCH PROJECTS USING NGS TECHNOLOGY

***6.1***: Is the subject of secondary use of material and / or data explained on your REB(s)' documents (templates, guidelines, other)?

(Please, mark all that apply)

Consent form templates / Guidelines / Other

If secondary use is explained, is it presented:

a) Under a title or subtitle indicating clearly the subject (for instance: “Future use of samples“)

b) Under a title or subtitle that does not mention future (secondary) use (for instance: “Confidentiality”)

c) Other

***6.2***: Would you prefer the subject of secondary (future) use be in a separate section (own title/subtitle) on the consent form? Yes / No / I don't know / Other

***6.3***: In your REB(s) consent form/guidelines is the explanation of secondary use of material presented separately from an explanation of secondary use of the generated genetic data?

Yes / No / I don't know / Other

***6.4***: Do you consider explanations to research participants about the difference between secondary use of genetic material and secondary use of genetic data to be: (Please, mark all that apply)

Yes No I don't know Other

important for participants’ informed consent

limiting to research projects

Other: (Please, explain)

***6.5***: On the consent documents that you oversee, can research participants choose to indicate, among different options, their own preference regarding secondary use of sample or genetic data generated (for example, secondary use for related diseases, for unrelated diseases, etc)

Yes No I don't know

Material only /Data only /Material and data together as one entity / Material and data as two different entities

Comments:

**7 - MANAGEMENT OF RETURN OF RESULTS**

MANAGEMENT OF RETURN OF INDIVIDUAL RESULTS (IR) AND INCIDENTAL FINDINGS (IF)

***7.1***: Returning individual research results and incidental findings from genetic/genomic studies implies financial support and human resources to carry out the endeavour. From your perspective, who should fund, organize and be responsible for communicating individual research results and incidental findings from genetic/genomic studies?

(Please, mark all that apply)

FUND ING / ORGANIZING / RETURN RESULTS

IR _ IF

Institution

Granting agency

Research Team

REB

I don’t know

Other; (If Other, please explain:)

***7.2***: In the past 12 months, have you noticed an increase in the number of discussions at board meetings concerning the return of research results? (Please, mark all that apply)

Yes No I don't know

Aggregated Research Results

Individual Research Results

Incidental Findings

Did the majority of those discussions relate to genetic/genomic studies?

Comments:

***7.3***: Do you anticipate more discussions at board meetings concerning…

Yes No I don't know

Aggregated Research Results

Individual Research Results

Incidental Findings

Comments:

***8 - DEMOGRAPHICS***

***8.1***: Gender: Male Female

***8.2***: Geographic area:

***8.3***: How did you learn about this survey?

Direct email REB administrator REB Colleague Professional network Other
