# Supplementary material for: Impact of Next Generation Sequencing on the Organization and Funding of Returning Research Results: Survey of Canadian Research Ethics Boards Members
Source: PLoS One. 2016 May 11;11(5):e0154965. doi: 10.1371/journal.pone.0154965 (PMC4868059; doi:10.1371/journal.pone.0154965)
Supplement: S1 Table — (DOCX) [file pone.0154965.s002.docx]

**Conveying Genetic Incidental Findings: Age of onset and age of participants and its relation to the severity of a health condition^[[1]](#footnote-1)^ - Part I**

**S1 Table:**

**IF a LIFE THREATENING condition can NOT be prevented**

Do you agree with offering research participants the communication of incidental findings for this type of condition? (N=67)

**Participant is young :**

| **Age of onset** | **No** | | **Yes** | | **I don't know** | |
| --- | --- | --- | --- | --- | --- | --- |
|  | **N** | **%** | **N** | **%** | **N** | **%** |
| Early age of onset (childhood, adolescence) | 4 | 12.1 | 26 | 78.8 | 3 | 9.1 |
| Onset in young adulthood (20 to 34 years of age) | 3 | 8.8 | 29 | 85.3 | 2 | 5.9 |
| Onset in adulthood (35 to 54 years of age) | 3 | 9.1 | 28 | 84.8 | 2 | 6.1 |
| Late age of onset (55 plus) | 3 | 9.1 | 28 | 84.8 | 2 | 6.1 |

**Participant is adult :**

| **Age of onset** | **No** | | **Yes** | | **I don't know** | |
| --- | --- | --- | --- | --- | --- | --- |
|  | **N** | **%** | **N** | **%** | **N** | **%** |
| Onset in young adulthood (20 to 34 years of age) | 0 | 0,0 | 30 | 96,8 | 1 | 3,2 |
| Onset in adulthood (35 to 54 years of age) | 0 | 0,0 | 32 | 100,0 | 0 | 0,0 |
| Late age of onset (55 plus) | 0 | 0,0 | 31 | 100,0 | 0 | 0,0 |

**IF a LIFE THREATENING condition CAN be prevented**

Do you agree with offering research participants the communication of incidental findings for this type of condition? (N=66)

**Participant is young :**

| **Age of onset** | **No** | | **Yes** | | **I don't know** | |
| --- | --- | --- | --- | --- | --- | --- |
|  | **N** | **%** | **N** | **%** | **N** | **%** |
| Early age of onset (childhood, adolescence) | 4 | 7.1 | 49 | 87.5 | 3 | 5.4 |
| Onset in young adulthood (20 to 34 years of age) | 3 | 5.5 | 51 | 92.7 | 1 | 1.8 |
| Onset in adulthood (35 to 54 years of age) | 5 | 9.3 | 46 | 85.2 | 3 | 5.6 |
| Late age of onset (55 plus) | 7 | 13.2 | 44 | 83.0 | 2 | 3.8 |

**Participant is adult :**

| **Age of onset** | **No** | | **Yes** | | **I don't know** | |
| --- | --- | --- | --- | --- | --- | --- |
|  | **N** | **%** | **N** | **%** | **N** | **%** |
| Onset in young adulthood (20 to 34 years of age) | 0 | 0,0 | 46 | 97.9 | 1 | 2.1 |
| Onset in adulthood (35 to 54 years of age) | 0 | 0,0 | 48 | 100.0 | 0 | 0.0 |
| Late age of onset (55 plus) | 0 | 0,0 | 45 | 95.7 | 2 | 4.3 |

**IF a NON-LIFE THREATENING condition can NOT be prevented**

Do you agree with offering research participants the communication of incidental findings for this type of condition? (N=64)

**Participant is young :**

| **Age of onset** | **No** | | **Yes** | | **I don't know** | |
| --- | --- | --- | --- | --- | --- | --- |
|  | **N** | **%** | **N** | **%** | **N** | **%** |
| Early age of onset (childhood, adolescence) | 3 | 11.1 | 22 | 81.5 | 2 | 7.4 |
| Onset in young adulthood (20 to 34 years of age) | 2 | 7.7 | 23 | 88.5 | 1 | 3.8 |
| Onset in adulthood (35 to 54 years of age) | 2 | 7.7 | 22 | 84.6 | 2 | 7.7 |
| Late age of onset (55 plus) | 2 | 7.7 | 22 | 84.6 | 2 | 7.7 |

**Participant is adult :**

| **Age of onset** | **No** | | **Yes** | | **I don't know** | |
| --- | --- | --- | --- | --- | --- | --- |
|  | **N** | **%** | **N** | **%** | **N** | **%** |
| Onset in young adulthood (20 to 34 years of age) | 0 | 0,0 | 26 | 100.0 | 0 | 0.0 |
| Onset in adulthood (35 to 54 years of age) | 0 | 0,0 | 26 | 100.0 | 0 | 0.0 |
| Late age of onset (55 plus) | 0 | 0,0 | 26 | 100.0 | 0 | 0.0 |

**IF a NON-LIFE THREATENING condition CAN be prevented**

Do you agree with offering research participants the communication of incidental findings for this type of condition? (N=61)

**Participant is young :**

| **Age of onset** | **No** | | **Yes** | | **I don't know** | |
| --- | --- | --- | --- | --- | --- | --- |
|  | **N** | **%** | **N** | **%** | **N** | **%** |
| Early age of onset (childhood, adolescence) | 3 | 6.7 | 40 | 88.9 | 2 | 4.4 |
| Onset in young adulthood (20 to 34 years of age) | 3 | 6.8 | 40 | 90.9 | 1 | 2.3 |
| Onset in adulthood (35 to 54 years of age) | 4 | 9.1 | 37 | 84.1 | 3 | 6.8 |
| Late age of onset (55 plus) | 8 | 18.2 | 34 | 77.3 | 2 | 4.5 |

**Participant is adult :**

| **Age of onset** | **No** | | **Yes** | | **I don't know** | |
| --- | --- | --- | --- | --- | --- | --- |
|  | **N** | **%** | **N** | **%** | **N** | **%** |
| Onset in young adulthood (20 to 34 years of age) | 0 | 0,0 | 39 | 97,5 | 1 | 2,5 |
| Onset in adulthood (35 to 54 years of age) | 0 | 0,0 | 40 | 97,6 | 1 | 2,4 |
| Late age of onset (55 plus) | 0 | 0,0 | 39 | 95,1 | 2 | 4,9 |

1. Percentages do not total 100% because respondents could check all that apply. Not all the participants who answered “Yes” responded to the detailed questions on age of participants and age of onset. [↑](#footnote-ref-1)
