# Supplementary material for: Impact of Next Generation Sequencing on the Organization and Funding of Returning Research Results: Survey of Canadian Research Ethics Boards Members
Source: PLoS One. 2016 May 11;11(5):e0154965. doi: 10.1371/journal.pone.0154965 (PMC4868059; doi:10.1371/journal.pone.0154965)
Supplement: S2 Table — (DOCX) [file pone.0154965.s003.docx]

**Conveying Genetic Incidental Findings: Age of onset and age of participants and its relation to the severity of a health condition^[[1]](#footnote-1)^ - Part II**

**S2 Table:**

**If it was possible to return IF for a serious condition that could be prevented, depending on the chance in which case would you offer return IF?**

| Age of  onset  Chance | Early age | | Young adulthood | | Adulthood | | Late in life | | No | | I don’t know | |
| --- | --- | --- | --- | --- | --- | --- | --- | --- | --- | --- | --- | --- |
|  | **N** | **%** | **N** | **%** | **N** | **%** | **N** | **%** | **N** | **%** | **N** | **%** |
| 1% Chance  (n=51) | 16 | 31.4 | 21 | 41.2 | 19 | 37.3 | 17 | 33.3 | 19 | 37.3 | 10 | 19.6 |
| 10% Chance  (n=52) | 30 | 57.7 | 33 | 63.5 | 33 | 63.5 | 27 | 51.9 | 5 | 9.6 | 12 | 23.1 |
| 50% Chance  (n=55) | 39 | 70.9 | 43 | 78.2 | 42 | 76.4 | 40 | 72.7 | 1 | 1.8 | 10 | 18.2 |
| 90% Chance  (n=54) | 43 | 79.6 | 44 | 81.5 | 44 | 81.5 | 43 | 79.6 | 1 | 1.9 | 7 | 13 |

1. Percentages do not total 100% because respondents could check all that apply. Not all the participants who answered “Yes” responded to the detailed questions on age of participants and age of onset. [↑](#footnote-ref-1)
