# Supplementary material for: Impact of Next Generation Sequencing on the Organization and Funding of Returning Research Results: Survey of Canadian Research Ethics Boards Members
Source: PLoS One. 2016 May 11;11(5):e0154965. doi: 10.1371/journal.pone.0154965 (PMC4868059; doi:10.1371/journal.pone.0154965)
Supplement: S3 Table — (DOCX) [file pone.0154965.s004.docx]

**S3 Table: Genetic counseling**

| Does your REB (current and / or past) require researchers to offer genetic counseling in genetic/genomic projects that plan to return IRRs and/or IFs? (n*=57) | n | % |
| --- | --- | --- |
| Yes | 30 | 52.6 |
| No | 14 | 24.6 |
| Other | 13 | 22.8 |
| Does your REB (current and past) require researchers to add an explanation on the provision of genetic counseling on the consent forms? (n= 55) |  |  |
| Yes | 34 | 61.8 |
| No | 13 | 23.6 |
| Other | 8 | 14.5 |
| Do you consider an explanation on the provision of genetic counseling to be important for participants’ informed consent? (n= 56) |  |  |
| Yes | 51 | 91.1 |
| No | 5 | 8.9 |
| Is/are the institutions to which your REB (current and past) belongs helping to make genetic counseling available? (n=46) |  |  |
| Yes | 31 | 56.4 |
| No | 15 | 27.3 |

* n refers to number of respondents
