# Supplementary material for: Impact of Next Generation Sequencing on the Organization and Funding of Returning Research Results: Survey of Canadian Research Ethics Boards Members
Source: PLoS One. 2016 May 11;11(5):e0154965. doi: 10.1371/journal.pone.0154965 (PMC4868059; doi:10.1371/journal.pone.0154965)
Supplement: S4 Table — (DOCX) [file pone.0154965.s005.docx]

**S4 Table: Financial costs of returning IRRs and IFs and the role of REB members**

| **Position on a REB** | **IRRs (n=58)** | | **IFs (n=57)** | |
| --- | --- | --- | --- | --- |
|  | Yes (%) | No (%) | Yes (%) | No (%) |
| Chair | 45.5 | 54.5 | 45.5 | 54.5 |
| Jurist/Ethicist | 81.8 | 18.2 | 100 | 0 |
| Member of Community | 11.1 | 88.9 | 0 | 100 |
| Scientific Member | 28.8 | 71.4 | 30.8 | 69.2 |

IRR: individual research results

IFs: Incidental Findings

REB: research ethics board

N: number of respondents
